# Selection for tameness modulates the expression of heme related genes in silver foxes

**DOI:** 10.1186/1744-9081-3-18

**Published:** 2007-04-17

**Authors:** Julia Lindberg, Susanne Björnerfeldt, Morten Bakken, Carles Vilà, Elena Jazin, Peter Saetre

**Affiliations:** 1 Department of Evolution, Genomics and Systematics, Uppsala University, Norbyvägen 18D, S-752 36 Uppsala, Sweden; 2 Department of Animal and Aquacultural Sciences, Norwegian University of Life Science, P.O. Box 5003 N-1432 Aas, Norway; 3 Department of Physiology and Developmental Biology, Uppsala University, Norbyvägen 18A, S-752 36 Uppsala, Sweden

## Abstract

**Background:**

The genetic and molecular mechanisms of tameness are largely unknown. A line of silver foxes (*Vulpes vulpes*) selected for non-aggressive behavior has been used in Russia since the 1960's to study the effect of domestication. We have previously compared descendants of these *selected *(*S*) animals with a group of *non-selected *(*NS*) silver foxes kept under identical conditions, and showed that changes in the brain transcriptome between the two groups are small. Unexpectedly, many of the genes showing evidence of differential expression between groups were related to hemoproteins.

**Results:**

In this study, we use quantitative RT-PCR to demonstrate that the activity of heme related genes differ between *S *and *NS *foxes in three regions of the brain. Furthermore, our analyses also indicate that changes in mRNA levels of heme related genes can be well described by an additive polygenic effect. We also show that the difference in genetic background between the two lines of foxes is limited, as estimated by mitochondrial DNA divergence.

**Conclusion:**

Our results indicate that selection for tameness can modify the expression of heme related genes in canid brain regions known to modulate emotions and behavior. The possible involvement of heme related genes in behavior is surprising. It is possible that hemoglobin modulates the behavior of canids by interaction with CO and NO signaling. Another possibility is that hemorphins, known to be produced after enzymatic cleavage of hemoglobin, are responsible for behavioral alterations. Thus, we hypothesize that hemoglobin metabolism can be a functionally relevant aspect of the domestic phenotype in foxes selected for tameness.

## Background

Domestication of animals can be described as the process by which wild animals adapt to coexistence with humans and to the environment we provide. Behavioral adaptation to the human environment is achieved through genetic changes occurring over generations together with environmental stimulation and experience during an animals lifetime [1]. A common aspect of early domestication is strong artificial selection for animals showing weak defensive behavior and low aggression towards humans [1], resulting in tame behavior.

The domestic dog (*Canis familiaris*) originated from the domestication of wild gray wolves (*Canis lupus*) at least 15,000 years ago [2,3]. The molecular mechanisms responsible for the behavioral changes associated with domestication in dogs are not known. We have previously shown that the brain gene expression profile of domestic dogs has diverged from that observed in their wild ancestor the gray wolf, suggesting that behavioral selection during domestication has modified mRNA expression levels of genes with multiple functions [4]. However, from the comparison of domestic animals with their free-living wild ancestor it is not possible to separate phenotypic differences that have a genetic background from those that are caused by differences in the animal's external environment and experiences. We therefore decided to study domestication in another model where environmental conditions could be well controlled. In the 1960s D.K. Belyaev, the late director of the Institute of Cytology and Genetics in Novosibirsk, initiated a domestication experiment on farmed silver foxes, a color morph of the red fox (*Vulpes vulpes*), to test the hypothesis that physiological and morphological changes in the domestic dog could have resulted from selection for tameness alone [5]. In this experiment, that still continues, farmed foxes have been selected for non-aggressive behavior towards man for more than 40 generations. The selection has resulted in animals that not only are friendly towards humans, but also are skilled as dogs in communicating with people [6]. Moreover, developmental, morphological and neurochemical changes (including modulations of the serotonergic system and the activity of the hypothalamus-pituitary-adrenal (HPA) axis) are associated with the behavioral changes in the selected foxes [7-10].

We have previously compared the global brain gene expression of descendants of the *selected *(*S*) silver foxes with a group of *non-selected *(*NS*) foxes, raised under identical conditions in a Norwegian farm [11]. Our microarray results suggested that behavioral changes caused by selection for tameness may be associated with only limited changes in the brain transcriptome. Moreover, our results indicated that expression of several genes related to heme have been affected by behavioral selection [11]; as many as six of the 14 clones showing the largest expression differences coded for hemoproteins.

In our previous experiments, mRNA samples from foxes were cross hybridized to human cDNA microarrays. Although this is a well accepted method for comparing transcriptional profiles within species [12], it requires an additional step of identifying the orthologous genes in fox that were responsible for the signals. Few fox mRNA sequences are available in public databases. However, chromosomal organization is almost the same in dogs and red foxes [13,14], and the sequence homology may be as high as 99.5% for individual genes [15]. Consequently, in this study we used dog sequences to design primers for quantitative real-time PCR (qPCR) to compare the mRNA levels of five heme-related canine sequences in the brains of the original sample set of *S *and *NS *foxes. To determine whether the mode of inheritance is primarily additive, we included animals which resulted from crossing the two original lines. In addition, we assess the divergence in genetic background between the *S *and *NS *lines, by comparing the left domain of the mitochondrial DNA (mtDNA) control region.

## Results

### Estimation of mtDNA divergence between selected and non-selected farm foxes

**Figure 1:**
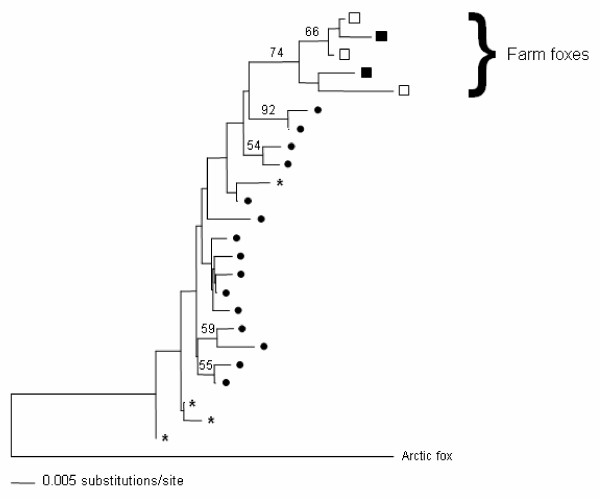
**Neighbor-joining phylogenetic tree based on mtDNA control region**. Sequences corresponding to the non-selected (*NS*) foxes (□), selected (*S*) foxes (■) and wild foxes from one population (*). Sequences of other wild foxes obtained from GenBank are indicated by a dot. Bootstrap support is indicated when higher than 50%.

To estimate the genetic variability and degree of differentiation between *S*, *NS *and *wild *foxes we compared 333 base pair (bp) sequences of the left domain of the mtDNA control region. These sequences were complemented with others corresponding to wild red foxes deriving from public databases (GenBank). The comparison of the mtDNA sequences were made for a total of 29 *NS*, 23 *S*, 23 *Cross*, 12 *wild *foxes and an additional 26 wild foxes retrieved from databases. Five different mitochondrial DNA haplotypes were observed in farm foxes, three of them in the *NS *and two in the *S *foxes. All hybrids (*Cross*) shared one of the *S *haplotypes as the females for line crosses always were from the *S *line and the mtDNA is maternally inherited. All sequences observed in farm foxes were very similar. Their pair-wise sequence divergence was lower than 3% in all cases and all clustered in one clade in a neighbor-joining tree (Figure 1). The genetic divergence between all farm fox haplotypes was similar to that observed in wild foxes sampled in a geographically limited region in Sweden. The four haplotypes observed in this wild population occupy different positions in the neighbor-joining tree. This indicates that the differences observed among farm animals (either selected or not) are comparable to the differences observed within one single natural population, and that *S *and *NS *lineages are closely related, with their haplotypes intermixed within one clade.

### Sequence similarity of heme-related genes between humans and canids

Since the expressed sequences in foxes should be very similar to sequences in dogs, we identified dog mRNA species that could have yielded one or more of the signals from the heme-related human clones on the arrays in our previous study [11] (Table 1). These dog mRNA species corresponded to five predicted dog genes, including four hemoglobins, namely, the beta (*HBB*) and epsilon (*HBE*) chains (located on canine chromosome 21), the alpha (*HBA*) and zeta (*HBZ*) chains (ESTs that mapped to canine chromosome 6), and the heme binding protein 1 gene (*HEBP1*) (located on canine chromosome 27). The canine mRNA sequence of *HBB *[GenBank accession number: LOC480784] and *HBE *[GenBank: LOC485256] were similar, as evident from a pair-wise blast (364 identities of 450 bp, e-value = 5e-55), and consequently, hybridization of either of the two mRNA species in foxes could have yielded the signals after hybridization to three human clones: 242594, 292528 and 239611. Similarly, the two canine ESTs, similar to human *HBA *[GenBank: CN003912] and *HBZ *[GenBank: DN366534], showed a high sequence similarity (207 identities of 270 bp, e-value = 4e-33), and either of the sequences expressed in fox could have yielded the signals on the two human clones 207558 and 248463. Finally, we could only identify one canine gene, [GenBank: LOC477690] which showed sequence similarities with the human clone 730346, encoding *HEBP1*.

**Table 1:** Sequence similarity between human clones giving signals of differential expression and dog mRNA sequence.

		**Dog mRNA sequence**				
**Human Clone**		LOC485256	LOC480784	CN003912	DN366534	LOC477690
ID	Symbol	(HBE)	(HBB)	(HBA^†^)	(HBZ^†^)	(HEBP1)
242594	HBG1	88%(242) 3e-78	79%(220) 1e-37	n.s.	n.s.	n.s.
292528*	HBG2	85%(426) 7e-127	81%(386) 5e-88	n.s.	n.s.	n.s.
239611	HBE	82%(175) 2e-32	81%(176) 8e-28	n.s.	n.s.	n.s.
207558	HBA2/HBZ	n.s.	n.s.	84%(309) 8e-75	73%(213) 8e-05	n.s.
248463	HBZ	n.s.	n.s.	88%(81) 8e-20	85%(155) 4e-36	n.s.
730346	HEBP1	n.s.	n.s.	n.s.	n.s.	76%(211) 3e-16

Local sequence alignment of heme related genes was carried out with BLAST (Bl2seq). Percentage identical nucleotides, number of nucleotides in alignment and e-values are listed. † = The canid hemoglobin alpha and zeta chains were retrieved from ESTs and have been annotated by comparison with human mRNA sequences. * = clone from array, sequenced in our laboratory. n.s. = no significant similarity found.

### Expression levels of hemoglobin related genes are modified by selection for tameness

**Figure 2:**
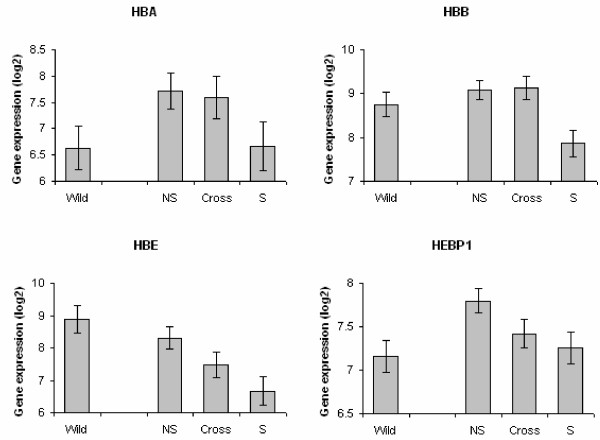
**Gene expression levels of heme related genes in fox brain**. Average mRNA levels of hemoglobin alpha (*HBA*), beta (*HBB*) and epsilon (*HBE*) chains, and heme binding protein (*HEBP1*) are shown for four groups of foxes, *Vulpes vulpes*. *Wild *= wild red foxes (N = 12), *NS *= non selected farm foxes (N = 17), *S *= farm foxes selected for tame behavior (N = 9), *Cross *= off-spring from crosses and back crosses of *NS *and *S *foxes (N = 12). Least square means and standard errors are given.

To examine which of the canine heme-related genes were differentially expressed in brains of selected silver foxes, and whether the mRNA levels showed evidence of a polygenic additive mode of inheritance, we designed primers based on canine sequences and quantified the mRNA levels corresponding to *HBB*, *HBE*, *HBA*, *HBZ *and *HEBP1 *in three regions of the brain in 9 *S *foxes, 17 *NS *and 12 *Cross *and 12 *wild*. Three hemoglobin genes, *HBE*, *HBB *and *HBA*, and the heme binding protein *HEBP1 *showed significantly lower mRNA levels in *S *foxes than in *NS *foxes in all brain regions (Figure 2). The response was similar in all three brain regions and there were no significant interactions between origin and region (Table 2). The difference between *S *and *NS *foxes was 3.1-fold for *HBE*, 2.3-fold for *HBB*, 2.2-fold for *HBA *and 1.5-fold for *HEBP1 *(Table 3). The mRNA levels of the foxes resulting from the crosses did not provide any strong evidence for a composite dominance effect (p-values > 0.13 for all genes, Table 3), and thus the major variation in mRNA levels between *S *and *NS *foxes appears to be due to a composite additive genetic effect. However due to the limited sample sizes, this study was underpowered to detect a moderate dominance effect. The power to detect a dominance effect between the two parental lines (d = a) were 0.21, 0.47, 0.40, and 0.19 for *HBA*, *HBB*, *HBE *and *HEBP1*, respectively. Consequently, it is possible that a true dominance effect of this size may have been overlooked.

**Table 2:** ANCOVA comparison of mRNA levels in three regions of the brain between groups of foxes.

	Num	HBA			HBB			HBE			HEBP1		
					
Effect	DF	DF	F ratio		DF	F ratio		DF	F ratio		DF	F ratio	
Origin	3	46	3.0	*	46	4.2	*	46	5.1	**	46	3.1	*
*Contrasts*													
1) S vs NS	1	46	4.1	*	46	10.4	**	46	8.7	**	46	5.6	*
2) C vs Par	1	46	0.3		46	2.4		46	0.2		46	0.8	
3) W vs F	1	46	3.8		46	0.0		46	7.8	**	46	2.2	
PCR Plate	1	89	231.6	***	90	66.2	***	88	10.5	**	89	21.8	***
Region	2	89	6.9	**	90	3.1	*	88	1.2		89	8.8	***
Origin*Region	6	89	0.6		90	1.0		88	1.1		89	1.4	
Ref. gene	1	89	131.3	***	90	122.8	***	88	24.9	***	89	156.4	***

For each gene the F-ratio is listed together with the corresponding degrees of freedom (DF) for the denominator. Statistical significance is marked with asterisk (*, P < 0.05; **, P < 0.01; ***, P < 0.001). The estimated differences in mRNA levels corresponding to the three contrasts are listed in table 3.

**Table 3:** Contrasts comparing the average gene expression levels in brain between groups of foxes.

Contrast	Gene	Fold Change	*p*-value
1) S vs NS^a^	HBE*	-3.1	0.005
	HBB	-2.3	0.002
	HBA	-2.2	0.048
	HEBP1*	-1.5	0.022
2) Cross vs parental^b^	HBE	-1.2	0.623
	HBB	1.4	0.132
	HBA	1.2	0.617
	HEBP1	-1.1	0.365
3) Wild vs Farm	HBE*	2.7	0.008
	HBB	1.0	0.853
	HBA	-1.9	0.059
	HEBP1*	-1.3	0.143

a = test of composite additive genetic effect.

b = test for composite genetic dominance effect.

* = previously reported in Lindberg *et al *[11].

The three contrasts tests the following mRNA differences: 1) difference between selected and non-selected farm foxes, 2) difference between off-spring from crossing and back crossing *S *and *NS *with the weighted average of the parental *S *and *NS *lines, and 3) difference between the average of farmed foxes (*S*, *NS *and *Cross*) and wild red foxes.

To estimate heme-related gene expression in blood cells, we also performed qPCR on dog blood, where all five examined transcripts were detected. Thus to evaluate the potential effect of blood perfusion on *HBE *and *HEBP1 *expression levels, we assumed that the average *HBA *and *HBB *levels (HB) could represent the maximum effect of blood perfusion caused by e.g. a systemic stress response. The *HBE *levels clearly increased with the normalized *HB *expression (p-value < 0.0001). However, even in presence of a perfusion effect *S *foxes had a 1.8-fold lower *HBE *mRNA levels than *NS *foxes (p-value = 0.031). The normalized *HB *level did not significantly affect the expression of *HEBP1 *(p-value = 0.18). Still if the perfusion effect was included in the statistical model, the difference in *HEBP1 *levels were reduced to 1.3-fold, suggesting that part of the *HEBP1 *difference between the *S *and *NS *foxes may be linked to the average *HB *mRNA levels. These results suggest that, while we cannot exclude a perfusion effect, part of the heme-related gene alterations is independent of this effect.

When we compared farmed foxes (*S*, *NS*, *Cross*) versus wild animals, we noted that *HBE *was the only gene to have mRNA levels that differed significantly between these two groups, with 2.7-fold higher levels in the wild foxes as compared to farmed (Table 3). This difference is difficult to explain as changes in environment and time post-mortem are confounded with the differences between the groups. Possible differences in *HBZ *levels could not be reliably quantified with our qPCR protocol because its mRNA levels were below the detection limit for almost half of the investigated samples.

## Discussion

We have previously shown that dramatic behavioral changes caused by selection for tameness may be associated with only limited changes in the brain transcriptome. In this study, we first evaluated whether differences in genetic background between *S *and *NS *farm foxes (rather than genetic differences due to selection for tameness) may be responsible for the observed expression differences, by estimating the mtDNA divergence between *S *and *NS *lines. Our results showed that the mtDNA lineages present in *S *and *NS *lines were similar to each other and did not cluster in reciprocally monophyletic groups. Although both *NS *and *S *foxes were likely to have reduced genetic diversity due to founding effects and subsequent drift, our results suggest that they are not likely to derive from wild populations that had been isolated from each other for a long time [16]. In fact, all farmed silver foxes are said to have originated from animals captured in Canada over a Century ago [17]. This could explain their differentiation from wild red foxes from Sweden and implies that *S *and *NS *foxes have a common origin.

Our previous microarray results suggested that heme-related genes in particular, show pronounced expression differences between *S *and *NS *foxes. Thus, in this study we used qPCR to quantify mRNA levels of five heme-related genes in *S*, *NS*, and foxes resulting from crossing the two parental lines, to determine whether we could find any evidence of an additive genetic effect underlying transcriptional differences between *S *and *NS *animals. We found that four of the five investigated genes, *HBA*, *HBB*, *HBE *and *HEBP1*, showed decreased expression levels in foxes selected for tameness compared to non-selected farm foxes. If multiple loci controlling gene expression have been under selective pressure, then we would expect that the expression variation between the two experimental populations (and the crosses between them) should primarily follow an additive mode of inheritance. For three of the genes, *HBA*, *HBE *and *HEBP1*, an additive genetic component did indeed appear to explain the observed expression variation. Thus, our results suggest that a selective pressure for tameness may result in direct or indirect modulation of heme and hemoglobin metabolism. However, for the fourth gene, *HBB*, the mRNA levels in foxes resulting from crossing and back-crossing the parental lines exceeded those of both the *S *and *NS *lines, and a simulation analysis revealed that moderate deviations from additivity could not be ruled out for any of the investigated genes given the limited power of the experiment.

The comparisons with animals in the wild are more difficult to interpret. However, from the comparison of *S *foxes with wild foxes it is clear that the abundance of each individual transcript does not predict tameness independently of context. These results are not surprising since we have previously shown that environmental effects together with adaptation to life in captivity have a much larger influence on brain gene expression in foxes, than selection for tameness [11]. It is possible that mRNA levels of heme related genes also are affected by the living conditions of foxes, and that mRNA levels in wild foxes have been further modified by mRNA degradation (since the *post mortem *interval in wild foxes was unknown). Thus strict interpretation of the comparisons of mRNA levels in wild and farmed foxes is not possible, due to the confounding effect of selection with systematic differences in environmental variables. Our results thus demonstrate the need for rigorous control of living environment and experimental conditions when studying the evolution of the brain transcriptome.

Four of the five investigated genes encode hemoglobin subunits. The *HBA*, *HBB*, *HBE *and *HBZ *genes encode globins (alpha, beta, epsilon and zeta) which, together with iron-containing heme groups, form hemoglobin subunits [18]. The fifth gene, *HEBP1*, encodes a monomeric and soluble protein that binds heme in both man and mouse [19]. *HEBP1 *is induced during erythroid differentiation and the protein is likely to be involved in utilization of heme for synthesis of heme proteins [20]. The coordinated response to selection for tameness in these genes suggests that heme metabolism and hemoglobin levels may have been modulated in the brains of tame animals.

In humans, hemoglobin alpha- and beta transcripts are known to be expressed in both leukocytes and reticulocytes of circulating blood [21,22]. In the normal human adult, alpha- and beta chains account for more than 98% of the total globin chains in blood [21]. As hemoglobin alpha- and beta also are the two dominating globins in fox blood [23], it is possible that the increased mRNA levels of these genes in *NS *foxes could be a manifestation of an increased perfusion in the brain as a direct consequence of a stronger stress response to human handling. However, *HBA *and *HBB *differences between *S *and *NS *foxes could only partly account for the observed decreased levels of *HBE *and *HEBP1 *in selected foxes. Thus, although a systemic response to stress may have contributed to the observed differences in levels of hemoglobin transcripts, our results suggest that selection may also have direct effects on the heme metabolism in the brain of tame animals. Possible consequences of an altered heme metabolism in the brain on tame behavior are discussed below.

Although involvement of hemoglobin genes in behavior is surprising, several observations support this possibility. First, a gene involved in behavior should be expressed in brain cells, and both hemoglobin α and β are expressed in neurons and glial cells [24]. Second, besides the well known role as an oxygen transport molecule, hemoglobin also binds carbon monoxide (CO) and nitric oxide (NO) with high affinity, and acts as an extra-cellular scavenger of these signaling molecules in the mammalian brain [20]. Evidence from *in vivo *studies demonstrates that CO produced from the enzymatic cleavage of heme, has important biological functions in the control of the stress response of mammals [25,26]. Nitric oxide also modulates the neuroendocrine responses and behavior that is linked to serotonin and the HPA-axis [27-29]. A third evidence for a potential role of hemoglobin in behavior is based in the knowledge that β-, κ-, δ- and ε-chains of hemoglobin also serve as the main precursors for hemorphins, a class of endogenous "non-classical" opioid peptides [30]. Hemorphins are naturally occurring peptides in brain, plasma and cerebrospinal fluid, with high binding affinity to μ- and δ-opioid receptors [30]. Although the function of hemoglobin in the brain is not known, hemorphins induce release of β-endorphin and dynorphin, endogenous opioids that are released in response to acute stress or pain [31,32]. From these results we postulate that hemorphins may also modulate stress responses in the fox. Similarly, it has been suggested that hemorphins produce dampening of exploratory and aggressive behavior in mice [33,34].

We have previously showed that genetic changes in behavior caused by selection for tameness may be associated with only limited changes in the brain transcriptome [11]. In this study, we present evidence indicating that selection for tameness has modulated heme and hemoglobin metabolism in the brain, and that the (additive) mode of inheritance of mRNA expression is similar to that of tame behavior. It can not be ruled out that part of the observed response of heme related transcripts is the result from a systemic stress response. However, our results indicate that selection has also had direct effects on the heme metabolism in the brain of tame animals, and we hypothesize that heme-related genes are directly, or indirectly, involved in behavior related to tameness.

## Experimental Procedures

### Animals

Samples from two groups of farm silver foxes (*Vulpes vulpes*), *selected *(*S*) and *non-selected *(*NS*) for tame behavior, were used in this study. The selected (*S*) foxes were descendants from two male and three female Russian silver foxes that were imported to Norway in 1996 from the Institute of Cytology and Genetics at the Russian Academy of Sciences (Novosibirsk, Russia) and belonged to a line of foxes that had been selected for tame behavior for more than 40 generations [8]. The non-selected (*NS*) animals consisted of Norwegian farm-bred silver foxes that had not been selected for tame behavior. We also included animals from crossing (*Cross*) the two parental lines(*F1*) and fromback-crossing the *F1 *animals to the *NS *line. The silver foxes were kept at a farm belonging to the Norwegian University of Life Science (Ås, Norway) and housed in cages of standard dimensions in accordance with the regulations of the Norwegian Ministry of Agriculture and Food. Foxes of the *S *and *NS *lines were randomly located in cages within the same building, were treated in the same way, had equal amount and kind of interaction with humans and were fed simultaneously. The foxes were euthanized in a random order for all groups during two consecutive days during January 2004, and all brains were removed and frozen to -80°C within 20 minutes after death. Samples of wild red foxes (*wild*) from central Sweden were used for comparison. These animals were shot during fox hunts in February 2004 and submitted by hunters to the Swedish National Veterinary Institute for necropsy, where brains were collected and frozen to -80°C. This resulted in a wider variation in postmortem intervals, which might have affected transcript levels, along with several other environmental differences (e.g. living conditions, diet, method used to kill them). Sex and age of individual foxes used in this study can be found in Table S1 in the Supplementary Material (see Additional file 1). All foxes studied died for other reasons than their participation in this study.

### Tissue samples and isolation of nucleic acids

Amygdala, frontal lobe and hypothalamus were morphologically identified with the support of a pathologist at the Swedish National Veterinary Institute. Samples from the three regions were processed as previously described [4,11]. Briefly, frozen tissue was homogenized in TRIzol^®^, and RNA was extracted according to the manufacturers' instructions (Invitrogen™, Life Technologies). About 10–30 μg of tissue were digested over night at 37°C in 500 μl of Laird's buffer (0.1 M Tris, 5 mM EDTA, 0.2 M NaCl, 7 mM SDS, adjusted to pH 8.5) with 0.3 mg proteinase K. DNA was extracted using a modified phenol/chloroform protocol [35]. For some samples, DNA was extracted from the TRIzol^® ^leftovers after RNA isolation using the PureGene^® ^DNA kit (Gentra systems). The concentrations of extracted RNA and DNA were measured using a NanoDrop^® ^ND-1000 instrument (NanoDrop Technologies, USA).

### MtDNA control region amplification and sequencing

For the mtDNA analysis, DNA was extracted from 29 *NS*, 23 *S*, 23 *Cross *(16 *F1 *+ 7 backcrosses) and 12 wild foxes. The left domain of the mitochondrial control region was PCR-amplified and sequenced using the primers, Thr-L 15926 5'-CAA TTC CCC GGT CTT GTA AAC C-3' and DL-H 16340 5'-CCT GAA GTA GGA ACC AGA TG-3' [36]. The amplification was done in a reaction volume of 10 μl, which included 1 × HotStar buffer, 0.5 μM of each primer, 0.2 mM dNTP, 2.25 mM MgCl_2_, 0.45 U HotStarTaq^® ^and 20 ng DNA. A negative control was included in the PCR reaction to check for contamination. The PCR profile included an initial denaturation step at 95°C for 7 min followed by 35 cycles of amplification (denaturation at 95°C for 1 min, annealing at 50°C for 2 min and extension at 72°C for 1 min and 30 s) and a final extension at 72°C for 10 minutes. PCR products were then purified with ExoSAP-IT (Amersham Biosciences). Sequencing of the purified PCR products was done on a MegaBACE™ 1000 (Amersham Biosciences) following protocols and using chemistry recommended by the manufacturer. The forward and reverse sequences for each fragment were aligned with Sequencher 4.1.4 (Gene Codes Corporation, Ann Arbor, MI) and consensus sequences were constructed.

### Phylogeny reconstruction

A neighbor-joining tree was built with sequences corresponding to the farm foxes and wild foxes. Additionally to these, 26 red fox sequences were obtained from public databases as well as one arctic fox (*Alopex lagopus*) that was used as outgroup. A neighbor-joining phylogenetic tree was obtained with PAUP 4.0b10 [37] using the Kimura 2-parameter model of sequence evolution, with a gamma shape parameter a = 0.5. The support for the tree topology was evaluated with 1000 bootstrap pseudo-replicates.

### Real-Time RT-PCR assay

Messenger-RNA was isolated from amygdala, hypothalamus and frontal lobe from a total of 50 foxes, including 17 *NS*, 9 *S*, 12 *Cross *(6 *F1 *+ 6 backcross) and 12 wild foxes as described previously [11]. In brief, each sample was extracted with PolyATtract^® ^mRNA Isolation Systems (Promega) and converted to cDNA using TaqMan^® ^Gold RT-PCR Kit (Applied Biosystems). Preparations of 21 μl qPCR reactions using SYBR^® ^Green PCR Core Reagents kit (Applied Biosystems) were added to plates where 4 μl of all 150 samples (50 individuals × 3 brain regions) were randomly distributed on two 96-well plates according to a balanced incomplete block design with respect to origin (*S*, *NS, Cross *and *wild*), sex and tissue. The sequences of the human clones corresponding to *HBG1*, *HBG2*, *HBE*, *HBZ/HBA1*, *HBZ*, and *HEBP1 *present in the arrays used for the study of Lindberg et al. (2005) were used for BLAST searches of the dog genome, and the homologous dog sequences were used to design qPCR primers. Primers were also designed for the reference genes *GAPDH *(Glyceraldehyde-3-phosphate dehydrogenase) and *ACTB *(Actin, beta); all primer sequences are available in the Supplementary Material (see Additional file1). The qPCR reactions were run on an ABI PRISM^® ^7000 Sequence Detector System (Applied Biosystems) using the default thermal profile; 50°C for 2 minutes and 95°C for 10 minutes, followed by 40 cycles of amplification with denaturation at 95°C for 15 seconds and annealing/extension at 60°C for 1 min.

### Real-Time RT-PCR data analysis

The qPCR data was analyzed with the following mixed ANCOVA model:

*y*_*jkli *_= *μ *+ *P*_*i*_+ *O*_*j*_+ *F*(*O*)_*jk*_+ *R*_*l*_+ (*OR*)_*jl*_+ *β G*_*ikl *_+ *ε*

where *y*_*jkli *_refers to the mRNA expression in l^th ^brain region of fox jk, *P *to the i^th ^PCR-plate, *O *to the j^th ^group of origin (*wild*, *S*, *NS*, *Cross*), *F *the k^th ^fox within group j, *R *to the l^th ^region of the brain, *OR *to the interaction between the j^th ^group and the l^th ^region, *G *to the reference gene expression in region l of fox jk and *β *to the regression coefficient. Initially, both age and sex were included in the statistical analysis but the effect of these parameters was not shown to be significant. When included as potential confounders, neither age nor sex affected the expression difference between *S *and *NS *foxes, and consequently these variables were excluded from the final model. In the analysis, the differences between origin (*S*, *NS*, *Cross*, *wild*) is assessed using the between-animal variability, whereas the effects of region and the interaction between region and origin is assessed using the variability of samples within animal. We used three contrasts to: 1) compare the average mRNA levels of *S *and *NS *foxes (corresponding to an additive genetic effect), to 2) test the dominance deviation by comparing the mRNA levels of the foxes resulting from crosses to the weighted average of the parental lines (5/8 *NS *+ 3/8 *S*, which represents the expected value for the crossed foxes under an additive model), and 3) to test the difference between wild foxes and the average of all farmed foxes. To account for variation in mRNA quantity, the geometric mean of the expression of the two reference genes *ACTB *and *GAPDH *[38] were included as the covariate (*G*) in the model [39]. The analysis was carried out in Proc Mixed, SAS v8.2. A modified version of the above linear model was used for power analysis of a genetic model including a composite additive (a) and a composite dominance (d) effect. That is, mRNA levels of the target genes were simulated, replacing "O" value in the formula above with "a" for *S *foxes, "-a" for *NS *foxes, "d" for F1 animals, and "0.5 d" for backcrosses. The interaction terms OR were dropped from the model as there were little evidence for any interactions between origin and region (Table 2). For the simulations, observed reference genes expression was used together with estimated effects for: "a", "P", "R", "O_wild_", and "*β*," and the dominance effect "d" was set to equal "a". The random effects "F_jk_" and "ε_ijkl_", were drawn from two normal distributions (0, σ_F_) and (0, σ_ε_) respectively, again using estimates of the between and within animal variance components from our data. For each gene 10 000 simulations were carried out and the power to detect an additive effect was defined as the fraction of simulations in which the contrast *S *versus *NS *was significant at α = 0.05. The power to detect dominance was defined as the fraction of simulations in which the contrast Cross vs. (5/8 *NS *+ 3/8 *S*) was significant at α = 0.05.

To examine if an increased blood perfusion could be the main driver of the observed mRNA differences between *S *and *NS *foxes, we assumed that the average *HBA *and *HBB *levels (*HB*) may reflect the maximum effect of blood perfusion. Since the average *HB *level was correlated to the reference gene expression (r^2 ^= 0.52, slope = 1.0 p-value < 0.0001), we normalized the *HB *levels prior to the analysis. This was done by regressing the average *HB *expression onto the total reference gene expression and storing the residuals as the normalized *HB *expression. This orthogonalized *HB *expression is positive when the amount of *HB *is larger than expected from the reference gene expression, and is negative when the average *HB *amount is lower than expected from the reference gene expression. The *HB *mRNA level was then included as an additional covariate in the original model, and thus the expression difference of *HBE *and *HEBP1 *was evaluated taking the maximum perfusion effect into account.

## Competing interests

The author(s) declare that they have no competing interests.

## Authors' contributions

JL participated in sample collection and preparation, carried out the expression analysis and participated in drafting of the manuscript. SB participated in sample collection and preparation, and carried out the mtDNA analysis. MB coordinated the sample collection. CV participated in the design of the study and the phylogeny reconstruction. EJ conceived of the study and participated in its design, coordination and manuscript drafting. PS participated in the design of the study, performed the statistical analysis and coordinated the preparation of the manuscript. All authors read and approved the final manuscript.

## Supplementary Material

Additional File 1 Primer sequences and additional data on individual foxes. The file can be viewed with Adobe Reader [40] Click here for file
