# Supplementary material for: Selection for tameness modulates the expression of heme related genes in silver foxes
Source: Behav Brain Funct. 2007 Apr 17;3:18. doi: 10.1186/1744-9081-3-18 (PMC1858698; doi:10.1186/1744-9081-3-18)
Supplement: Additional File 1 — Primer sequences and additional data on individual foxes. The file can be viewed with Adobe Reader [40] [file 1744-9081-3-18-S1.pdf]

Supplementary material

Primer sequences

HBA Fwd 5’ TGACCTCCAAGTACCGTTAAGCT
HBA Rev 5’ CTCAGACTTTATTCCAACATCAGGAA
HBB Fwd 5’ CTCACCACTTTGGCAAAGAATTC
HBB Rev 5’ ACCAGCCACCACCTTCTGAT
HBE Fwd 5’ GACACCTTTGCTGAGCTAAGTGAA
HBE Rev 5’ CATGTTGCCTAGAAGCTTGAAGTTC
HBZ Fwd 5’ GGACCATCATCCTGTCCATGT
HBZ Rev 5’ AGGGCCTCGGTGCCAAT
HEBP1 Fwd 5’ AGTCCCTATTTCCTTTGCTGTGTT
HEBP1 Rev 5’ TGGAATCCGGAACCAGACTT

Table S1. Foxes used for real-time RT-PCR.

ID	Age[1]	Sex[2]	Origin[3]
V227/04	2	M	wild
V230/04	2	M	wild
V231/04	2	M	wild
V247/04	1	M	wild
V296/04	1	M	wild
V297/04	1.5	F	wild
V299/04	3	F	wild
V301/04	1	M	wild
V302/04	6	F	wild
V226/04	3	M	wild
V232/04	4	M	wild
V300/04	6	M	wild
300022	4	F	S
302034	2	F	S
303120	1	M	S
303121	1	M	S
303122	1	M	S
396517	8	M	S
303100	1	M	S
303101	1	F	S
303104	1	F	S
001004	3	F	NS
003017	1	M	NS
003021	1	F	NS
003023	1	M	NS
003024	1	M	NS
003029	1	M	NS
003033	1	F	NS
003087	1	F	NS
003088	1	M	NS
003025	1	M	NS
003027	1	M	NS

003091	1	M	NS
003092	1	F	NS
003028	1	F	NS
003078	1	F	NS
003079	1	F	NS
003093	1	F	NS
303105	1	M	F1
303107	1	M	F1
303108	1	M	F1
303110	1	M	F1
303109	7	M	F1
399066	5	F	F1
003063	1	M	BC
303034	1	M	BC
303035	1	M	BC
303036	1	M	BC
303038	1	F	BC
303039	1	F	BC

[1] = given in years
[2] = F (female), M (male)
[3] = wild, S (selected), NS (non-selected), F1 (cross between NS and S), BC (cross between F1 and NS).
